# Diagnostic Accuracy of a Rapid Influenza Test for Pandemic Influenza A H1N1

**DOI:** 10.1371/journal.pone.0010364

**Published:** 2010-04-28

**Authors:** Aubree Gordon, Elsa Videa, Saira Saborío, Roger López, Guillermina Kuan, Angel Balmaseda, Eva Harris

**Affiliations:** 1 Division of Epidemiology, School of Public Health, University of California, Berkeley, California, United States of America; 2 John E. Fogarty International Center, National Institutes of Health, Bethesda, Maryland, United States of America; 3 Sustainable Sciences Institute, Managua, Nicaragua; 4 Departamento de Virología, Centro Nacional de Diagnóstico y Referencia, Ministry of Health, Managua, Nicaragua; 5 Centro de Salud Sócrates Flores Vivas, Ministry of Health, Managua, Nicaragua; 6 Division of Infectious Diseases and Vaccinology, School of Public Health, University of California, Berkeley, California, United States of America; Singapore Immunology Network, Singapore

## Abstract

**Background:**

With the current influenza A H1N1 pandemic (H1N1pdm), it is extremely important that clinicians can quickly and accurately identify influenza cases.

**Methodology/Principal Findings:**

To investigate the performance of the QuickVue Influenza A+B rapid test, we conducted a prospective study of the diagnostic accuracy of the QuickVue Influenza A+B test compared to real-time reverse transcriptase-polymerase chain reaction (RT-PCR) for influenza A H1N1pdm in Nicaraguan children aged 2 to 14 years. Rapid test sensitivity and specificity compared to real-time RT-PCR were 64.1% (95% CI 53.5, 73.9) and 98.3% (95.0, 99.6), respectively. Agreement between the two tests was 86.4% (95% CI 81.7, 90.3), and kappa was calculated to be 0.67 (95% CI 0.56, 0.76). Performance of the rapid test varied by day of presentation, with a sensitivity of 41.7% (95% CI 22.1, 63.4) for samples from children presenting on the day of symptom onset and a sensitivity of 72.1% (95% CI 59.9, 82.3) for samples from children presenting one or more days post-symptom onset.

**Conclusions/Significance:**

We found that the rapid test performed with moderate sensitivity and high specificity. Test performance varied by day of onset, with lower sensitivity on the day of symptom onset.

## Introduction

With the current H1N1 influenza pandemic and potential shortage of antiviral medications, especially in developing countries, it is important that physicians rapidly and accurately diagnose influenza A H1N1 pandemic (H1N1pdm) cases. Rapid point-of-care diagnostic tests that can be performed in under 15 minutes provide a significant time advantage over other laboratory-intensive influenza testing methods. Rapid tests aid in clinical decision-making, reduce inappropriate antibiotic use, and decrease emergency department visit time [1], [2], [3]. Additionally, they allow for testing in resource-limited settings where equipment, reagents, and highly trained laboratory personnel are not always available.

The performance of rapid tests in detecting seasonal influenza A and B has been reported in numerous studies [1], [4], [5], [6], [7], [8]. Rapid tests have been shown to have lower accuracy than the reference tests of viral culture and reverse transcriptase polymerase chain reaction (RT-PCR), with reported sensitivities of rapid tests ranging from 27% to 90% and specificities ranging from 86% to 100% [1], [4], [5], [6], [7], [8]. The reported performance of rapid tests for pandemic influenza A H1N1 has varied, with sensitivities ranging from 10% to 75% [9], [10], [11], [12], [13], [14].

The objective of this study was to investigate the diagnostic accuracy of the QuickVue Influenza A+B rapid test for influenza A H1N1pdm compared to real-time RT-PCR in children in Nicaragua.

## Methods

### Ethics Statement

This study was approved by the institutional review boards at the Nicaraguan Ministry of Health and the University of California, Berkeley. Written informed consent was obtained from the parents or guardians of all participants, and assent was obtained from those participants aged 6 and over.

### Study Design

The Nicaraguan Influenza Cohort Study is an ongoing prospective, community-based cohort study of influenza in ∼3,800 children 2–14 years old in Managua, Nicaragua. Healthy children were recruited into the study through house-to-house visits in neighborhoods surrounding the study health center, Health Center Sócrates Flores Vivas (HCSFV). Each year, new 2-year-old participants are enrolled. At enrollment, parents agree to bring their child to study physicians at first sign of illness. Data from all medical visits are collected onto standardized study instruments. The case definition for testing used in this analysis was any participant with fever or history of fever and with cough or sore throat of <5 days duration. A nasal respiratory specimen was collected using the swab provided in the kit for rapid testing. A second sample, consisting of nasal and throat Dacron swabs, was collected simultaneously for use in real-time RT-PCR testing. The rapid tests were performed immediately on-site at the HCSFV clinical laboratory according to manufacturer's instructions. The clinical laboratory staff had previous experience collecting nasal and throat swabs. Clinical laboratory staff received an additional 1.5 hours of training on influenza, the principles of the rapid test, and how to collect samples for and perform the rapid test.

Samples for RT-PCR testing were kept at 4°C and transported to the National Virology Laboratory at the Ministry of Health within 72 hours. QiaAmp Viral RNA isolation kits were used to extract RNA from the viral transport media. Real-time RT-PCR was performed according to standard protocols developed by the Centers for Disease Control and Prevention (CDC) [15] and using probes and primers provided by the CDC. The complete protocol, along with primer and probe sequences, can be found online at www.who.int/csr/resources/publications/swineflu/CDCrealtimeRTPCRprotocol_20090428.pdf. All RT-PCR testing was performed in the Nicaraguan National Virology Laboratory by personnel trained to perform the protocol by the CDC. Personnel performing RT-PCR assays were blinded both to the rapid test results and to the clinical presentation of the patients.

Outcome measures of this study are sensitivity, specificity, positive predictive value (PPV), and negative predictive value (NPV). Test agreement was calculated using the kappa statistic. Sub-analyses were performed to examine rapid test performance in children meeting the CDC definition of influenza-like illness (fever≥37.8°C with cough or sore throat) and to analyze the performance of the rapid test by day after symptom onset. To investigate any effect of including the samples from 13 children who contributed two samples a subanalysis limited to the first sample contributed by a participant was performed. No significant difference was found when second samples were excluded, and thus all results reported in this study include the second samples. All statistical analyses were performed using STATA 10.1 (StataCorp, College Station, Texas).

## Results

Two hundred and sixty-seven samples were collected from 254 cohort participants meeting the case definition; thirteen children contributed a sample from two separate illness episodes. The participants were aged 2 to 14 years (median 6.9 years). Of the 267 cases, 72 (27%) presented on the day of developing symptoms, 146 (55%) presented 1 day after onset of symptoms, 22 (8%) presented 2 days post-symptom onset, and 27 (10%) presented 3 or more days after developing symptoms. One hundred and thirty six cases met the CDC definition of influenza-like illness. Two samples tested positive for seasonal influenza by RT-PCR and were excluded from the analysis. In total, 92 (35%) samples tested positive for H1N1pdm by RT-PCR. The rapid test detected 59 (64%) of the RT-PCR influenza-positive samples. The sensitivity and specificity of the rapid test compared to RT-PCR were 64.1% (95% CI 53.5, 73.9) and 98.3% (95.0, 99.6), respectively (Table 1). Agreement between the two tests was 86.4% (95% CI 81.7, 90.3) and kappa was calculated to be 0.67 (95% CI 0.56, 0.76). Sub-analysis of the 136 cases that met the CDC definition of influenza-like-illness did not result in significant changes in test performance, nor did including the two seasonal influenza cases (data not shown).

**Table 1:** Performance of the QuickVue Influenza A+B Rapid Test for Influenza A H1N1pdm, Managua, Nicaragua, 2009.

	Number of Positive Samples	Sensitivity		Specificity		Positive Predictive Value		Negative Predictive Value	
	(%)	(%)	(95% CI)	(%)	(95% CI)	(%)	(95% CI)	(%)	(95% CI)
All cases	92 (35)	64.1	(53.5, 73.9)	98.3	(95.0, 99.6)	95.2	(86.5, 99.0)	83.7	(77.9, 88.5)
Day of symptom onset	24 (33)	41.7	(22.1, 63.4)	97.9	(88.9, 99.9)	90.9	(58.7, 99.8)	77.0	(64.5, 86.8)
≥1 day post symptom onset	68 (35)	72.1	(59.9, 82.3)	98.4	(94.3, 99.8)	96.1	(86.5, 99.5)	86.6	(79.9, 91.7)

Sub-analyses examining test performance by day of onset were performed. Among children who presented on the day of symptom onset, rapid test sensitivity and specificity compared to RT-PCR were 41.7% (95% CI 22.1, 63.4) and 97.9% (95% CI 88.9, 99.9). Test agreement was 79.2% (95% CI 68.0, 87.8), and kappa was 0.45 (95% CI 0.19, 0.64). Diagnostic accuracy of the rapid test was higher for children presenting ≥1 days after symptom onset, with a sensitivity of 72.1% (95% CI 59.9, 82.3) and a specificity of 98.4% (95% CI 94.3, 99.8). Agreement between the tests was 89.1% (95% CI 83.8, 93.1), and kappa was 0.75 (95% CI 0.62, 0.83).

## Discussion

We found that the QuickVue Influenza A+B rapid test performed moderately well, with an intermediate sensitivity and a high specificity in comparison to the CDC real-time RT-PCR assay for influenza A H1N1pdm. Furthermore, we found that test performance varied depending on how many days after symptom onset the patient presented at the clinic.

Several studies have been published on the performance of the QuickVue Influenza A+B rapid test in detecting influenza A H1N1pdm, with reported sensitivities ranging from 51 to 75% [11], [12], [13], [14]. Our sensitivity estimate of 64.1% falls within the range of previously reported sensitivities and is very similar to the estimate of a sensitivity of 62.7% from the Suntarattiwong et al. study in Thai children [11]. In this same Nicaraguan cohort, we have previously evaluated the performance of the rapid test for seasonal influenza A in children compared to RT-PCR. In that analysis, the rapid test was found to have a sensitivity of 65.2% (95% CI 58.5, 71.4) and a specificity of 99.1% (95% CI 98.3, 99.6) which is very similar to the results found for H1N1pdm in this study [16]. Likewise, in our previous study, we found a significantly lower sensitivity of the rapid test for seasonal influenza in samples collected on the day of symptom onset compared to samples collected one or two days following symptom onset.

In this study, we used separate samples for the rapid test and the RT-PCR. Although this method could result in some discordant results, we do not feel that this is a limitation of the study. Rather, in order to accurately assess the performance of the rapid test, the swab provided with the test and recommended by the manufacturer must be used. Likewise, for RT-PCR the current standard in Nicaragua and many countries is a combined nasal and throat swab. Furthermore, the rapid test swab is unusable for other diagnostic purposes once the swab is inserted into the testing solution. Thus, by adhering to the current, recommended sample collection procedures for each test respectively, this comparison allows us to assess the diagnostic accuracy of the QuickVue rapid test compared to RT-PCR in real-world conditions. In addition, the use of the two different sample types may explain the specificity of less than 100% observed in the study.

One limitation of this study is that families participating in the Nicaraguan Influenza Cohort Study are encouraged to bring their child in for medical care at the very first sign of illness, which results in a significant proportion of children presenting on the day of symptom onset in this study (27%). Since we found that rapid test performance was lower on the day of symptom onset, using this population may result in a conservative overall estimate of the rapid test performance. To address this issue, we performed sub-analyses by day of onset. For the general clinical population, the performance of the test may be closer to our estimate of a sensitivity of 72.1% and specificity of 98.4%, which we calculated for children who presented one or more days after symptom onset.

Our findings support that the QuickVue rapid test performs moderately well in the detection of influenza A H1N1pdm. Of note, we found that the test performs with low sensitivity on the day of symptom onset.

## References

[1] Poehling KA, Zhu Y, Tang YW, Edwards K (2006). Accuracy and impact of a point-of-care rapid influenza test in young children with respiratory illnesses.. Archives of Pediatrics and Adolescent Medicine.

[2] Bonner AB, Monroe KW, Talley LI, Klasner AE, Kimberlin DW (2003). Impact of the rapid diagnosis of influenza on physician decision-making and patient management in the pediatric emergency department: results of a randomized, prospective, controlled trial.. Pediatrics.

[3] Abanses JC, Dowd MD, Simon SD, Sharma V (2006). Impact of rapid influenza testing at triage on management of febrile infants and young children.. Pediatric Emergency Care.

[4] Uyeki TM (2003). Influenza diagnosis and treatment in children: a review of studies on clinically useful tests and antiviral treatment for influenza.. Pediatric Infectious Diseases Journal.

[5] Hurt AC, Alexander R, Hibbert J, Deed N, Barr IG (2007). Performance of six influenza rapid tests in detecting human influenza in clinical specimens.. Journal of Clinical Virology.

[6] Agoritsas K, Mack K, Bonsu BK, Goodman D, Salamon D (2006). Evaluation of the Quidel QuickVue test for detection of influenza A and B viruses in the pediatric emergency medicine setting by use of three specimen collection methods.. Journal of Clinical Microbiology.

[7] Grijalva CG, Poehling KA, Edwards KM, Weinberg GA, Staat MA (2007). Accuracy and interpretation of rapid influenza tests in children.. Pediatrics.

[8] Uyeki TM, Prasad R, Vukotich C, Stebbins S, Rinaldo CR (2009). Low sensitivity of rapid diagnostic test for influenza.. Clinical Infectious Diseases.

[9] Ginocchio CC, Zhang F, Manji R, Arora S, Bornfreund M (2009). Evaluation of multiple test methods for the detection of the novel 2009 influenza A (H1N1) during the New York City outbreak.. Journal of Clinical Virology.

[10] CDC (2009). Performance of Rapid Influenza Diagnostic Tests During Two School Outbreaks of 2009 Pandemic Influenza A (H1N1) Virus Infection—Connecticut, 2009.. MMWR CDC Surveillance Summaries.

[11] Suntarattiwong P, Jarman RG, Levy J, Baggett HC, Gibbons RV (2009). Clinical Performance of a Rapid Influenza Test and Comparison of Nasal Versus Throat Swabs to Detect 2009 Pandemic Influenza A (H1N1) Infection in Thai Children.. Pediatric Infectious Disease Journal.

[12] Kok J, Blyth CC, Foo H, Patterson J, Taylor J (2009). Comparison of a Rapid Antigen Test with Nucleic Acid Testing During Co-circulation of Pandemic Influenza A/H1N1 2009 and Seasonal Influenza A/H3N2.. Journal of Clinical Microbiology.

[13] Watcharananan S, Kiertiburanakul S, Chantratita W (2009). Rapid influenza diagnostic test during the outbreak of the novel influenza A/H1N1 2009 in Thailand: An Experience with Better Test Performance in Resource Limited Setting.. Journal of Infection.

[14] Vasoo S, Stevens J, Singh K (2009). Rapid antigen tests for diagnosis of pandemic (Swine) influenza A/H1N1.. Clinical Infectious Diseases.

[15] Dawood FS, Jain S, Finelli L, Shaw MW, Lindstrom S (2009). Emergence of a novel swine-origin influenza A (H1N1) virus in humans.. New England Journal of Medicine.

[16] Gordon A, Videa E, Saborio S, Lopez R, Kuan G (2009). Performance of an influenza rapid test in children in a primary healthcare setting in Nicaragua.. PLoS ONE.

